# Exploring new subgroups for irritable bowel syndrome using a machine learning algorithm

**DOI:** 10.1038/s41598-023-45605-2

**Published:** 2023-10-28

**Authors:** Elahe Mousavi, Ammar Hassanzadeh Keshteli, Mohammadreza Sehhati, Ahmad Vaez, Peyman Adibi

**Affiliations:** 1 Medical Image and Signal Processing Research Center, School of Advanced Technologies in Medicine, Isfahan University of Medical Sciences, Hezar Jerib Street, PO Box 8174673461, Isfahan, Iran; 2 Department of Bioinformatics, School of Advanced Technologies in Medicine, Isfahan University of Medical Sciences, Isfahan, Iran; 3 Department of Internal Medicine, Integrative Functional Gastroenterology and Hepatology Research Center, School of Medicine, Isfahan University of Medical Sciences, Isfahan, Iran

**Keywords:** Data mining, Functional clustering, Machine learning, Gastroenterology

## Abstract

Irritable bowel syndrome (IBS) is a complicated gut-brain axis disorder that has typically been classified into subgroups based on the major abnormal stool consistency and frequency. The presence of components other than lower gastrointestinal (GI) symptoms, such as psychological burden, has also been observed in IBS manifestations. The purpose of this research is to redefine IBS subgroups based on upper GI symptoms and psychological factors in addition to lower GI symptoms using an unsupervised machine learning algorithm. The clustering of 988 individuals who met the Rome III criteria for diagnosis of IBS was performed using a mixed-type data clustering algorithm. Nine sub-groups emerged from the proposed clustering: (I) High diarrhea, pain, and psychological burden, (II) High upper GI, moderate lower GI, and psychological burden, (III) High psychological burden and moderate overall GI, (IV) High constipation, moderate upper GI, and high psychological burden, (V) moderate constipation and low psychological burden, (VI) High diarrhea and moderate psychological burden, (VII) moderate diarrhea and low psychological burden, (VIII) Low overall GI, and psychological burden, (IX) Moderate lower GI, and low psychological burden. The proposed procedure led to the discovery of new homogeneous clusters in addition to certain well-known Rome sub-types for IBS.

## Introduction

Irritable bowel syndrome (IBS) is one of the disorders of gut–brain interaction (DGBI) that is characterized by abdominal pain with changes in stool frequency and/or form and relation to defecation^1^. Based on a recent global study on the prevalence of DGBI, the prevalence of IBS ranged from 3 to 5% in most countries^2^. IBS, as a diverse condition with no identifiable anatomical and biochemical abnormalities, is characterized by distinct etiologies that generate diverse symptom combinations in different subgroups of patients^3,4^. However, no single biomarker can currently capture all of the pathophysiologic pathways that may cause IBS^1,3^. The co-occurrence of IBS with other DGBI is very high^5–8^, and the presence of extra-intestinal symptoms is also common in people with IBS^9^. Personality traits and psychological factors are key components of the biopsychosocial model of IBS and contribute to the brain-gut axis' functioning and dysregulation^10–12^.

The Rome III diagnostic criteria defined four subgroups by considering the evidence of the differences in bowel habits in the preponderance of IBS patients. Constipation‐predominant (IBS‐C), diarrhea‐predominant (IBS‐D), a mix of constipation and diarrhea (IBS‐M), and un-subtyped IBS (IBS‐U) are the well-known subgroups of IBS^13^. More stringent Rome IV criteria applied minor changes to these subgroups by defining the groups according to predominant bowel habits on the days with abnormal bowel movements^14^.

However, bowel habit fluctuation over time^15^, the existence of only two completely separable groups (i.e., IBS-C and IBS-D), the neglect of other common and associated factors besides bowel habits^16^, disregarding overlaps with other DGBI^5,17^, and incomplete satisfaction with the current treatments^18^, are all presented as concerns with the current classification of IBS in the literature^19–21^.

The mentioned points could be summed up in the question of whether it is possible to find more homogenous subgroups for IBS based on more various factors. A few recent studies, initiated by Polster et al., in 2017, have tried to answer this question using different sets of variables and procedures^9,20–24^.

In this study, we aimed to investigate the hypothesis of the existence of novel clusters (subgroups) of IBS patients, according to the symptoms of upper and lower gastrointestinal (GI), personality traits, somatic, and psychological scores. Thus, we applied machine learning methods for the clustering of patients. The current study is an endeavor to follow up on recent studies to provide stable, reproducible, discriminative, and conceptually meaningful clusters based on various related factors. This process could be followed by further experiments on the investigation of biomarkers and treatments in new homogenous groups.

## Methods

### The study population

The current study is a part of the Study on Epidemiology of Psychological Alimentary Health and Nutrition (SEPAHAN), a cross-sectional study conducted in Iran in 2010^25^. Self-administered questionnaires were used to collect demographic, GI symptoms, and psychological data from participants using a multistage random cluster selection method. To identify the patients with FGIDs, including IBS, the modified Persian version of the Rome III questionnaire was utilized^26^. During the questionnaire validation, it was found that distinguishing among the options used in the original Rome III questionnaire was difficult for the participant. Thus, a modified 4-point Likert scale (i.e., never or rarely, sometimes, often, always) was employed. Instead of questioning the onset of symptoms more than six months prior to the evaluation, the presence of each symptom in the previous three months was assessed^25^. Patients with IBS were identified according to the Rome III criteria. The criteria were defined based on the recurrent abdominal pain or discomfort at least sometimes in the previous 3 months associated with two or more of the following criteria: (1) improvement with defecation at least sometimes; (2) pain onset associated with a change in stool frequency; and (3) pain onset associated with a change in form (appearance) of stool, at least sometimes^25,27^. The protocol of the SEPAHAN study was approved by the Research Committee of our university and more details on the data set can be found in the SEPAHAN protocol paper^25^. All methods for data gathering were carried out in accordance with relevant guidelines and regulations. All study participants provided informed written consent before study enrollment^25^.

### GI symptom and psychological factors

For investigating the GI symptoms, the Rome III questionnaire, and for exploring psychological distress, anxiety, depression, and personality traits, the Iranian validated version of the General Health Questionnaire (GHQ12)^28^, the Hospital Anxiety and Depression Scale^29^, and the NEO Five-Factor Inventory (NEO-FFI) questioners^30^, were utilized, respectively. The maximum anxiety and depression scores are 21, while the GHQ12 range is 0–12. The GHQ-12 questionnaire covers various aspects of emotional and psychological well-being, using items such as the ability to concentrate, the quality of sleep, the capacity to play a useful role, decision-making abilities, stress levels, problem-solving skills, enjoyment of normal activities, the ability to cope with difficulties, etc.^28^. NEO-FFI covers the five main dimensions of personality, including neuroticism, extraversion, openness, agreeableness, and conscientiousness. Each factor has a range of 0–48, which is determined by summing up 12 items on a 5-point Likert scale. The psychosomatic symptom checklist^31^ was also utilized to assess the somatic symptoms, including headaches, backaches, asthma, shortness of breath, insomnia, feeling exhausted, stiffness, heart palpitation, joint pain, eye pain, dizziness, feeling shivering, flushing, and high blood pressure. The somatic symptom frequency score was calculated by summing up the 14 questions on a four-point rating scale (ranging from 0 to 42).

### Statistical methods

We used a machine-learning approach to explore the subgroups of individuals in our study population. Unsupervised learning or clustering algorithms are robust and automatic tools for determining a set of samples in such a way that samples in the same group (so-called cluster) are more similar (in some sense) to each other than to those in other groups (clusters). Distance or similarity of individuals could be defined from a variety of perspectives. Choosing an appropriate distance/similarity based on the nature of the data is a critical step in clustering that can lead to desirable results. We used our in-house developed method, Generalized Unified Distance Metric for Mixed-type data in combination with the Spectral clustering method (GUDMM-S)^32^, to cluster IBS patients, taking into account the ordinal and nominal nature of the GI questionnaire, as well as continuous scores obtained for psychological factors. GUDMM-S evaluates the relationships between variables in addition to imposing the distributional information of various types of variables. It is already known that there is an association between psychological and GI symptoms in IBS patients^4,33–35^. Thus, using the proposed procedure, the relationship between variables for the clustering of the patients has been considered.

To determine the number of clusters, various internal validation indices have been introduced in the literature. We evaluated the CVNN^36^, and S-Dbw indices^37^, which could be used to establish a balance between separation and compactness of clusters, by assessing their values throughout a range of cluster numbers. Furthermore, to evaluate the stability of the clusters, clustering results were examined by random sub-sampling of the population with different rates^38,39^.

The non-parametric analysis of variance (Kruskal–Wallis) test was used to assess the differences in variables between clusters, and the effect sizes were determined by ${\epsilon }^{2}$. Pairwise comparisons of clusters using the post hoc Conover test with Holm correction were also performed after the Multiple Comparison Test (MCT). In addition to the MCT, the comparison of each cluster vs. the rest of the samples was performed by the Mann–Whitney U test and Cliff's Delta effect size.

By adjusting each variable's cluster mean to the population mean, z-values for each variable were calculated. In a radar plot, variables are placed in the environment of a circle, and each variable has its own axis. According to the mean values of each variable in a cluster, its length changes on the corresponding axis. While the zero central circle shows the mean of the variables in the whole population, the distance of the second internal circle from the central circle equals 0.5 units of the standard deviation (σ) of each variable in the whole population. This distance grows for other circles accordingly. As a result, the variables become more dominant as one moves away from the zeros circle. The radar plot of all input variables for each cluster could be used to characterize each cluster visually.

To define the values of variables qualitatively (i.e., high, moderate, and low) in the final interpretation of the clusters, we used both the radar plots and the results of statistical tests. The values of more than 0.5σ from the zero central circle or having the p-values of < 0.001 and an effect size of more than 0.3 were used to define a variable as "high" in a cluster. In the same way, for values of variable placed in the central circle, the variable's level was considered “low”, and the range in this between was defined as “moderate”.

### Ethics approval and consent to participate

The ethical approval of the SEPAHAN study was approved by the Medical Research Ethics Committee of Isfahan University of Medical Sciences (#189069, #189082, and #189086). All study participants provided informed written consent before study enrollment. The data used for this study were fully anonymized. All methods for data gathering were carried out in accordance with relevant guideline and regulations.

## Results

### Characteristics of the study population

We analyzed the data of 988 individuals who fulfilled the Rome III criteria for the diagnosis of IBS. Patients were 19–69 years old (36.01 ± 7.17 years), and 623 (63%) of them were female. Forty-two percent of the patients had functional heartburn (FHB), and 32.18% had functional dyspepsia (FD). The distribution of subtypes identified by the Rome III criteria is 334 (33.8%), 202 (20.44%), 185 (18.7%), and 267 (27%) for IBS-C, IBS-D, IBS-M, and IBS-U, respectively. The mean values of depression, anxiety, GHQ12, somatization score, and NEO-FFI, were $7.5\pm 3.5$, $5.4\pm 4$, $3.1\pm 3.1$, $11.6\pm 5.9$, and ($21.7\pm 7.4, 27.9\pm 6.4, 24.5\pm 4.9, 30.7\pm 5.7, 35.7\pm 6.5$), respectively. A full list of GI variables and their abbreviation codes is represented in Table 1.

**Table 1:** Full list of the GI symptoms and their abbreviated codes.

Code	Variable	Code	Variable	Code	Variable
AP	Abdominal pain	IE	Sensation of incomplete evacuation	R	Regurgitation
AO	Anorectal obstruction	LS	Very loose or watery stool	RAPCS	Relieve of abdominal pain by change in the body situation
B	Bloating	LT	Sensation of a lump in the throat	RAPD	Relieve of abdominal pain by defecation
CFSAP	Change in the frequency/form of stool during abdominal pain	L3DW	Less than 3 defecations in a week	RB	Rectal burning
CFSEP	Change in the frequency/form of stool during epigastric pain	MM	Manual maneuvers for defecation	RDAAP	Restriction of the daily activates due to the abdominal pain
DR	Difficulty relaxing during bowel movement	MS	Mucus in stool	REPD	Relieve of epigastric pain\burn by defecation
EPB	Epigastric pain or burning	M3D	More than 3 defecations in a day	REPE	Relieve of epigastric pain\burn by eating
ES	Early satiation	N	Nausea	RP	Rectal pain
FT	Food sticking in the throat	NCP	Non-cardiac chest pain	SD	Straining during defecation
FTH	Food sticking in the throat with heartburn	PF	Postprandial fullness	SG	Stomach growling
HB	Heartburn	PS	Pain during swallowing	SI	Stool incontinence
HS	Lumpy or hard stools	PUA	Pain is in the upper or side of the abdomen	U	Urgency

### Clustering analysis

**Figure 1:**
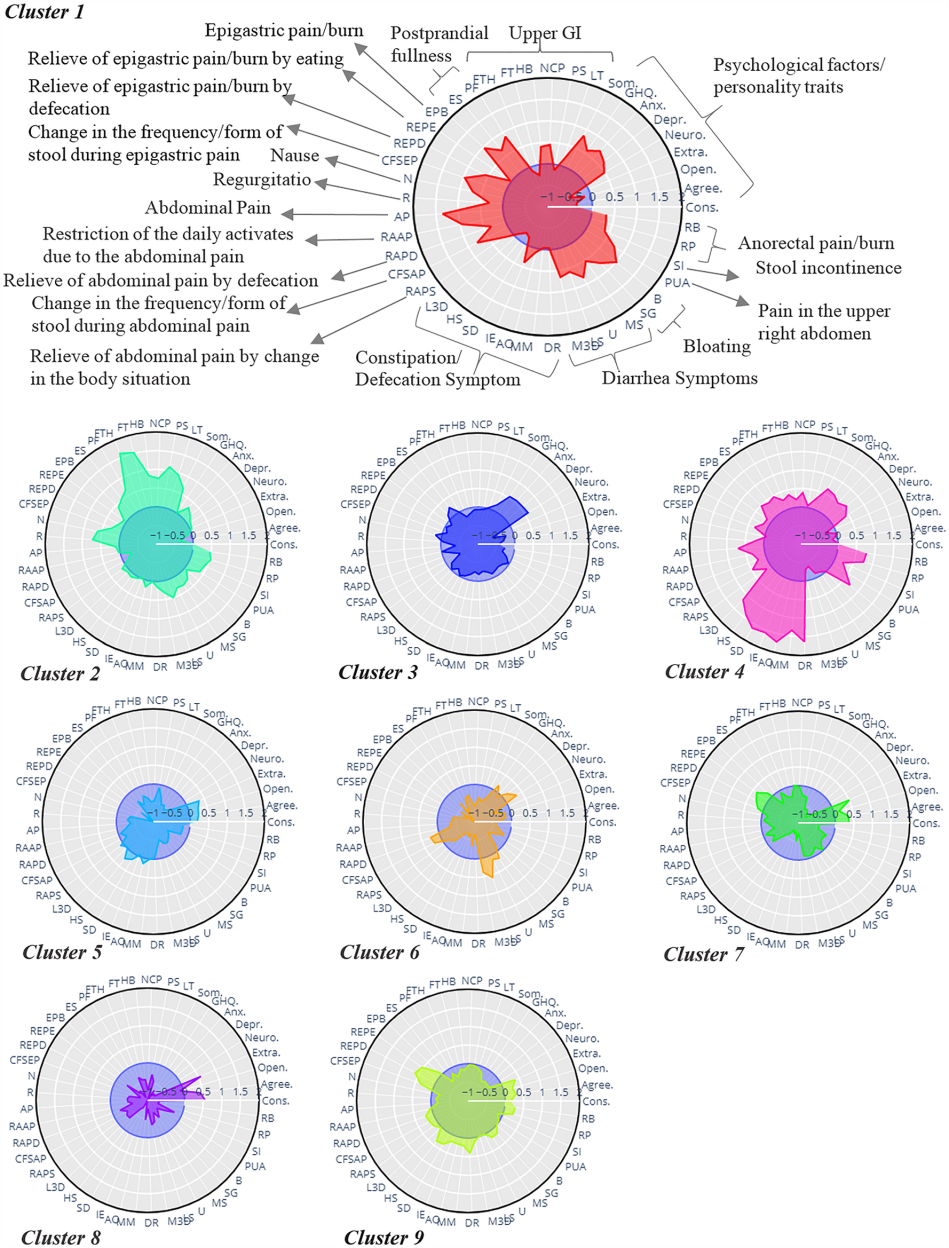
Profiles of the nine identified clusters. Cluster 1: high diarrhea, functional dyspepsia, and high psychological burden; Cluster 2: high upper GI, moderate lower GI, and psychological burden; Cluster 3: high psychological burden, and moderate overall GI; Cluster 4: high constipation, moderate upper GI, and high psychological burden; Cluster 5: constipation and low psychological burden; Cluster 6: high diarrhea and moderate psychological burden; Cluster 7: diarrhea and low psychological burden; Cluster, 8: low overall GI, and low psychological burden; Cluster 9: moderate lower GI, and low psychological burden.

**Figure 2:**
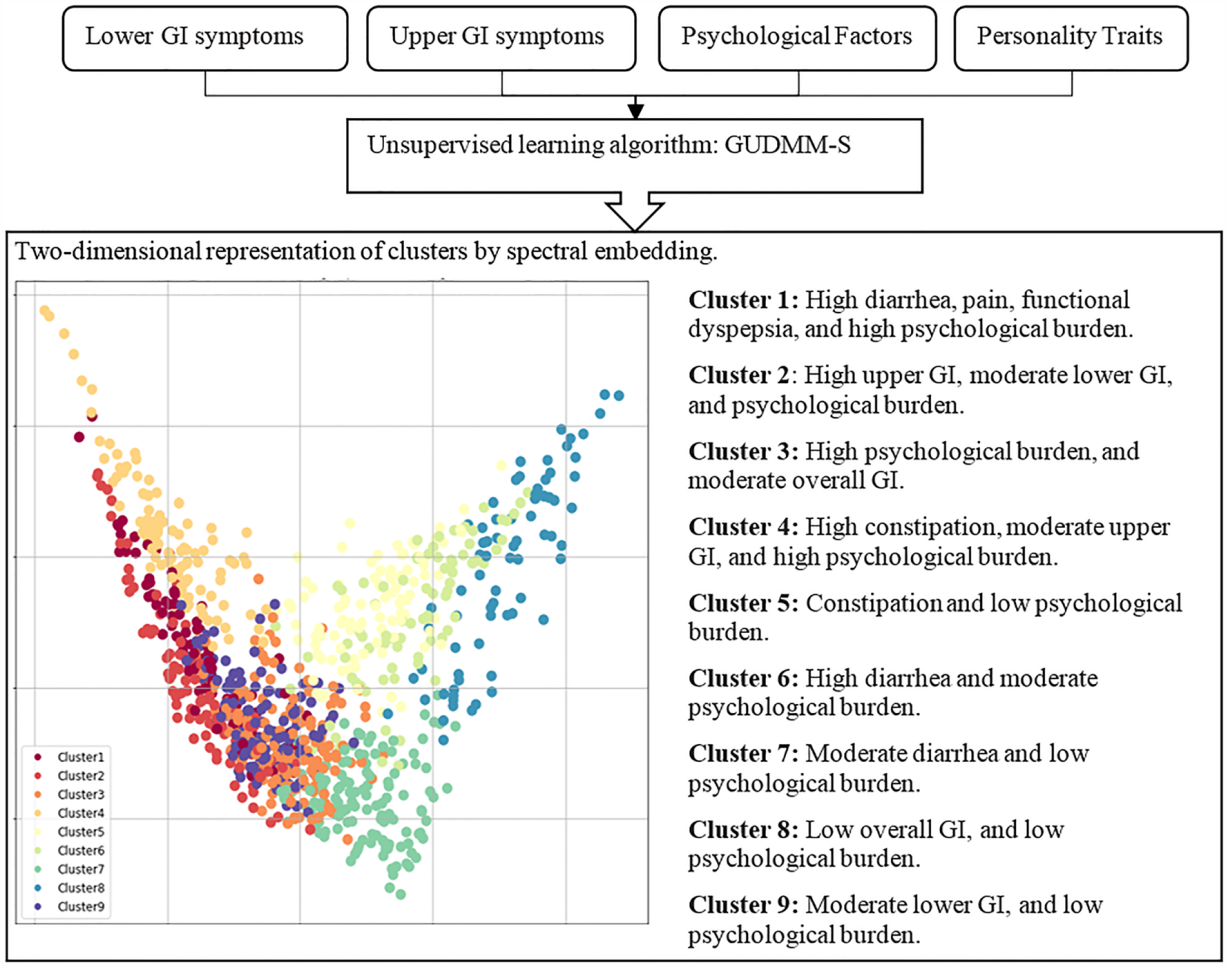
Cluster analysis of patients with IBS.

We used the CVNN and S-Dbw internal clustering evaluation indices to determine the number of clusters. The number of clusters corresponding to the minimum values of these indices suggests the best clustering results. We chose nine clusters based on the indices indicated in the supplementary Fig. 1. According to the expert knowledge and considering the predominance of specific symptoms or the existence of discriminant determinants, the profiles of 4 clusters (1, 2, 3, and 4) have a substantial difference from the average scores of the population and could be introduced as new subgroups, while the rest of the clusters had low/medium values and indicate the core of IBS. The radar plots of clusters are indicated in Fig. 1, while the flowchart of the proposed procedure and a brief description of clusters are summarized in Fig. 2. To illustrate the separation of clusters in a 2-dimensional space, we used the spectral embedding technique. All samples were projected on a new two-dimensional space for better visualization. The more compact each cluster is and the more separated from other clusters, the better the results of the clustering. However, it's worth noting that the clusters of IBS patients may not exhibit perfect separation due to the substantial overlaps in IBS patient profiles. The visualization of the identified cluster is exhibited in Fig. 2.

Cluster 1 is characterized by high abdominal and epigastric pain, diarrhea, postprandial fullness, bloating, chest pain, anorectal pain, as well as high psychological factors, neuroticism, GHQ, and somatic symptom score. According to the statistical test results indicated in Supplementary Table 2, all somatic symptoms are significantly higher than the other clusters except for asthma and blood pressure. Compared to the Rome III criteria, more than 70% of subjects in this cluster were identified as either IBS-D or IBS-M. More than 80% of the samples in this cluster reported abdominal pain usually or always and having the urgency of stool sometimes or more.

Cluster 2 is characterized by the dominance of upper GI symptoms, including the sensation of a lump in the throat (SLP), pain during swallowing (PS), non-cardiac chest pain (NCP), heartburn (HB), food sticking in the throat (FST), and food sticking in the throat with heartburn (FSTH). This cluster also exhibits symptoms of diarrhea and anorectal pain or burning, along with moderate levels of other factors such as depression, GHQ12, abdominal pain and its related symptoms, and hard stool. Additionally, it is associated with a high somatization score. Although GHQ, depression, anxiety, somatization, and neuroticism have a high positive correlation with each other and a negative correlation with four other personality traits in most clusters, in cluster 2, the somatic score is significantly higher than average in comparison to other psychological factors. The scores of all 14 somatic symptoms in this cluster are significantly higher than in other samples. Compared to the Rome III criteria, this cluster has a mix of individuals with IBS -M, IBS-C, or IBS-D. About 80% of this cluster’s members reported some times or more for upper GI symptoms and loos/watery stool.

Cluster 3 with moderate levels of all GI symptoms indicates high values for psychological factors, including depression, anxiety, neuroticism, and GHQ scores. Compared to the Rome III criteria, IBS-C or IBS-U were detected in more than 70% of individuals in this cluster.

Cluster 4 is a well-defined cluster with symptoms of constipation and abdominal and epigastric pain, as well as high psychological, GHQ, somatic, and neuroticism scores. Backache, heart palpitation and dizziness are the somatic symptoms that in this cluster indicate significantly higher values compared to other samples. Compared to the Rome III criteria, almost 75% of participants in this cluster were diagnosed with IBS-C. 80% of the samples suffered from straining during defecation, incomplete evacuation, and sensation of anorectal obstruction usually or always.

Two clusters, 1 and 4, in which high degrees of GI symptoms are observable, indicate the highest levels of diarrhea and constipation, respectively. Although both clusters show high epigastric and abdominal pain, in cluster 1, their levels are significantly higher. Furthermore, changes in the frequency or form of the stool with epigastric pain or burn and the restriction of daily activities due to abdominal pain in cluster 1 are also significantly higher than in cluster 4.

The rest of the clusters indicated low abdominal pain but with specific symptoms. Cluster 5 has moderate constipation and low psychological burden. The samples in cluster 6 indicated a high frequency of diarrhea and moderate psychological burden. Cluster 7 is characterized by moderate diarrhea and low psychological burden. Cluster 8 as a mild cluster denotes no significant GI symptoms and low psychological burden. Patients in Cluster 9 have moderate lower GI symptoms with low psychological burden.

The demographic information, distribution of other DGBI, and mean values of psychological factors in the identified clusters are summarized in Table 2. The results of the multiple comparison test and the post-hoc analysis of symptoms in the nine identified clusters, based on Kruskal–Wallis followed by Conover and Holm correction, have been summarized in Supplementary Table 1 and Supplementary Figs. 2 and 3. In addition to the MCT test of the input variables, the mean profile and the results of the MCT of extra-intestinal somatic symptoms in detail are also reported in Supplementary Fig. 1 and Supplementary Table 2, respectively.

**Table 2:** Characteristics of the identified clusters. The numbers in the parentheses represent the percent of samples in each cluster.

	Cluster1	Cluster 2	Cluster 3	Cluster 4	Cluster5	Cluster6	Cluster7	Cluster8	Cluster 9	P-value	E.S
# Samples	103	103	158	104	100	80	132	74	134		
# Female	75	54	106	80	66	51	71	39	81	0.0	0.02
Age (mean ± SD)	35.4 ± 7.1	37.1 ± 6.3	35 ± 6.8	36.9 ± 7.4	36.1 ± 7.2	35.8 ± 6.9	36.7 ± 7.4	34.9 ± 7.3	36.3 ± 7.7	0.14	0.01
IBS severity (median)	3	2	1	2	0	0	1	0	1	0.0	0.23
Seen a doctor in the last month	40 (39)	28 (27)	39 (25)	26 (25)	19 (19)	17 (21)	20 (15)	9 (12)	27 (20)	0.001	0.03
Seen a gastroenterologist in the last month	33 (32)	28 (27)	22 (14)	18 (17)	17 (17)	17 (21)	17 (13)	10 (14)	23 (17)	0.002	0.02
Depression (mean ± SD)	9.7 $\pm$ 3.7	8.2 $\pm$ 3.1	9.5 $\pm$ 2.8	9.6 $\pm$ 4	5.7 $\pm$ 2.5	8.1 $\pm$ 2.8	4.9 $\pm$ 2.2	4.9 $\pm$ 2.2	6.4 $\pm$ 2.4	< .0001	0.32
Anxiety (mean ± SD)	8.4 $\pm$ 4.7	6.7 $\pm$ 3.7	7.7 $\pm$ 3.5	7.4 $\pm$ 4.8	3.1 $\pm$ 2.5	5 $\pm$ 3.3	2.8 $\pm$ 2.2	2.1 $\pm$ 2.4	4.1 $\pm$ 2.7	< .0001	0.34
GHQ-12 (mean ± SD)	4.9 $\pm$ 3.4	3.1 $\pm$ 2.7	4.8 $\pm$ 3.1	5 $\pm$ 3.6	1.6 $\pm$ 2.2	3.7 $\pm$ 2.8	1.3 $\pm$ 1.7	1 $\pm$ 1.6	2 $\pm$ 2.5	< .0001	0.27
Somatization (mean ± SD)	16.5 $\pm$ 6	15.6 $\pm$ 5.8	12.5 $\pm$ 4.5	15.4 $\pm$ 6.6	8.7 $\pm$ 4.3	9.8 $\pm$ 4.1	8.2 $\pm$ 4.3	5.7 $\pm$ 3.3	10.7 $\pm$ 4.3	< .0001	0.35
FHB, N (%)	63 (61)	84 (82)	62 (39)	50 (48)	20 (20)	14 (18)	61 (46)	15 (20)	50 (37)	< .0001	0.14
FCP, N (%)	21 (20)	17 (17)	52 (33)	26 (25)	25 (25)	16 (20)	36 (27)	10 (14)	48 (36)	0.002	0.03
Globus, N (%)	11 (11)	0 (0)	14 (1)	9 (9)	7 (7)	5 (6)	12 (9)	5 (7)	15 (11)	0.11	0.01
Vomiting, N (%)	67 (65)	76 (74)	88 (56)	42 (40)	23 (23)	16 (20)	45 (34)	12 (16)	44 (33)	< .0001	0.14
FDG, N (%)	3 (3)	19 (18)	19 (12)	13 (13)	9 (9)	8 (10)	8 (6)	3 (4)	16 (12)	0.006	0.02
FD, N (%)	80 (78)	61 (59)	41 (26)	61 (58)	15 (15)	7 (9)	27 (21)	3 (4)	23 (17)	< .0001	0.3
PF, N (%)	64 (62)	48 (47)	26 (16)	50 (48)	13 (13)	6 (7)	18 (14)	1 (1)	15 (11)	< .0001	0.21
IBS-C, N (%)	16 (16)	22 (21)	55 (35)	76 (73)	64 (64)	6 (8)	20 (15)	8 (11)	67 (50)	< .0001	0.22
IBS-D, N (%)	39 (38)	21 (20)	24 (15)	2 (2)	5 (5)	42 (53)	43 (33)	13 (18)	13 (10)	< .0001	0.13
IBS-M, N (%)	30 (29)	44 (43)	21 (13)	20 (19)	10 (10)	12 (15)	6 (5)	2 (3)	40 (30)	< .0001	0.1
IBS-U, N (%)	18 (18)	16 (16)	58 (37)	6 (6)	21 (21)	20 (25)	63 (48)	51 (69)	14 (10)	< .0001	0.16

Functional heartburn, functional chest pain, functional dysphagia, functional dyspepsia, and postprandial fullness are abbreviated by FHB, FCP, FDG, FD, and PF, respectively.

In this study we did not use the IBS-Severity Scoring System (IBS-SSS) questionnaire^40^, but according to the summation of the severity of abdominal pain and bloating, three categories of mild, moderate, and high could be defined for the IBS-severity. Based on the median score, clusters 5, 6, and 8 showed mild severity, clusters 3, 7, and 9 represented moderate, and three clusters 1, 2, and 4 included patients with high severity. The notable point concerning this categorization is the severity of psychological factors. While Clusters 1 and 2 were identified with high levels of IBS-severity and psychological factors, Cluster 3 also indicated high levels of psychological factors, despite having moderate IBS severity. Further to this categorization, we investigated the presence of pain in the seven regions of the abdomen, including (1) epigastrium, (2) right lumbar, (3) umbilical, (4) left lumbar, (5) right iliac, (6) hypogastrium, and (7) left iliac regions. Based on the Kruskal–Wallis test, pain in the epigastrium and right lumbar regions is significantly different among the identified clusters (P-value = 0.001). More details of the segmental abdominal pain in nine identified clusters are indicated in Supplementary Figs. 5 and 6 and Supplementary Table 3.

**Figure 3:**
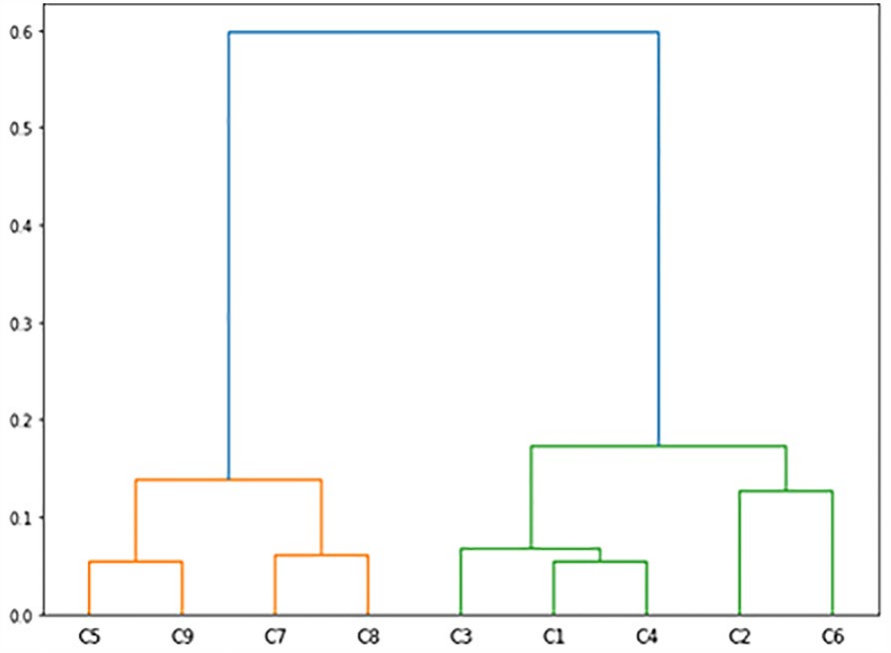
The hierarchical structure of the identified clusters (C1–C9) of IBS with regard to the distance of psychological factors.

Based on the level of psychological factors, identified clusters could be categorized as low or high psychological burdens. Clusters (1, 2, 3, 4, and 6) indicated a high psychological burden, and clusters (5, 7, 8, and 9) indicated low levels of psychological burden. The hierarchical structure of the identified clusters illustrated in Fig. 3, represents how the presence of psychological factors along with GI symptoms culminated in the separation of clusters into different levels of psychological factors, while in the classification of the Rome III criteria for diagnosis of IBS, these factors have been ignored.

To investigate the validity and stability of the results, in the current step, in the absence of other population studies, we sampled the current population with differing subsampling rates ranging from 90 to 98 percent. To ensure the consistency and stability of the clustering results, we conducted 10 iterations for each subsample. The clustering stability results were evaluated using Hungarian clustering accuracy (CA) across 10 iterations^38,39^. The average CA for various experiments was approximately 86%. Furthermore, the visually investigated profile of clusters did not indicate high differences among different subsampling experiments. These experiments were also performed for other numbers of clusters, and we found 9 clusters among the most stable results.

For future use of the introduced clusters, it is possible to predict the classes of patients using the known classifiers, e.g., support vector machine (SVM), which in our leave-one-out experiments resulted in an average accuracy of more than 80%.

## Discussion

Considering the shortcomings of the current classification in the treatment of IBS patients, we used a machine learning approach to cluster IBS patients based on the effects of factors other than stool consistency and frequency, including upper and lower GI symptoms, personality attributes, somatic, and psychological scores. In general, clustering is based on the similarity of individuals to each other. In addition to considering the nature of variables in the calculation of the similarity, we utilized our in-house developed method, the GUDMM-S clustering method, to imply the local dependency of variables. Based on internal validation indices of clustering (CVNN and S-Dbw), nine clusters of individuals with IBS were identified.

From the comparative perspective with Rome III sub-types, four clusters (4–5, 6, and 8) almost correspond to the IBS patients with IBS-C, IBS-D, and IBS-U, respectively. Of these four clusters, clusters (5, 6, and 8) are almost pure IBS and include no other DGBI. 20%, 21%, and 19% of samples with IBS-C, IBS-D, and IBS-U are in clusters 5, 6, and 8, respectively. These three clusters indicate mild abdominal pain and below-average values for most GI and psychological factors, except for cluster 6 (indication of pure IBS-D), which represents higher values of psychological factors, especially depression.

Further to the above categorization, considering the overall level of symptoms, four distinguishable clusters (1, 2, 3, and 4) indicate a high level of symptoms. In contrast, other clusters represent lower levels of symptoms and are near the core of IBS. In this categorization, clusters 1, 2, and 4, which showed the highest values of GI symptoms, indicated the highest coincidence of FD and IBS, with an overlap rate of 78%, 59%, and 60%, respectively. Cluster 3, which is composed of high values of psychological factors and a moderate level of GI symptoms, also indicated a 25% overlap with FD. When symptoms of other DGBI arise in individuals, other clusters can be identified based on a combination of more symptoms. The interaction of symptoms with each other could be identified as a contributor to the emergence of new IBS sub-groups. In other words, these findings highlight the requirements for more investigation on new clusters, which indicate the overlaps of IBS with other DGBI to understand the probabilistic pathological characteristics and relation with psychological factors.

In the current study, we included all the variables with the same weight, and we did not impose any prior information on the weights of the variables, i.e., considering GI and psychological factors in a unified framework. This methodology resulted in clusters with high psychological burden and moderate to high degrees of GI symptoms, whereas low psychological burden was observed in clusters with low to moderate GI symptoms. In other words, while the presence of high GI symptoms and high psychological factors has been observed simultaneously in the previous studies^41–43^ (as it was in clusters 1 and 4 in our study), by focusing on the profile of cluster 3, and 9 (which characterized with high psychological burden- average GI symptoms, and moderate GI symptoms- low psychological burden, respectively), it seems that incorporating psychological factors directly into the clustering procedure along with GI symptoms could be beneficial for defining more separable subgroups.

Recently, Polster et al. investigated the clustering of IBS patients using a Gaussian Mixture Model (GMM) based on the lower GI, somatic, and psychological symptoms^21,23^. They introduced seven subgroups of individuals, including constipation-low comorbidities; constipation-high comorbidities; diarrhea-pain low comorbidities; diarrhea-pain high comorbidities; mixed GI-high comorbidities; overall mild severity; and mild GI-high psychological. While in the current study, we only utilized the score of somatic symptoms and the whole upper GI factors, they included all somatic and no upper GI symptoms. However, a high overlap could be observed between the results of the two studies, especially between levels of lower GI and psychological factors. The first difference refers to the correspondence of two clusters, 1 and 6, with the subgroup diarrhea-high comorbidities, in which these two clusters discriminate against the presence of upper GI symptoms. Furthermore, the levels of diarrhea and psychological symptoms in cluster 1 (in which the upper GI symptoms were also dominant) were significantly higher. The other difference is related to the existence of cluster 9 (representing moderate lower GI-low psychological symptoms) that had no correspondence in the clusters defined in^23^.

The occurrence of seven distinct clusters was reported in another IBS clustering study conducted by Black et al. based on the lower GI, somatic symptoms, and psychological factors^20^. The characteristics of the seven reported clusters were: diarrhea predominance with high and low psychological factors; constipation predominance with high and low psychological factors; low overall GI symptoms with high and low psychological factors; and a cluster with high overall symptoms. Neglecting the variation of upper GI in clusters’ definitions, there is an overlap between these results and ours.

The other study on IBS clustering^9^, by considering IBS Quality of Life (QOL) and combining the severity and frequency of GI and somatic symptoms, introduced 4 clusters, including low symptoms-good QOL, low symptoms-moderate QOL, high symptoms-diarrhea-poor QOL, and high symptoms-low diarrhea-moderate QOL. Differences in the included factors and the identified number of clusters constrained the comparison of the results. However, this study also revealed that IBS patients can be divided into conceptually meaningful subgroups based on their GI and non-GI symptoms. A thorough comparison on the previous studies on clustering of IBS patients is indicated in Table 3.

**Table 3:** Comparison of the studies on IBS clustering.

Study	Year	Sample size	Criteria	Method	No. of clusters	Input variables	Results
Polster et al.^23^	2017	172	Rome III	Gaussian Mixture Model (GMM)	6	Lower GI, somatic, and psychological symptoms	Constipation with low/high comorbidities; diarrhea with low comorbidities; diarrhea and pain with high comorbidities, mixed GI with high comorbidities; a mix of symptoms with overall mild severity
Polster et al.^21^	2019	637	Rome III	GMM	7	IBS‐related GI symptoms extra intestinal somatic symptoms, psychological symptoms	constipation‐related; diarrhea‐related; mixed, and further distinguished by the presence or absence of non‐GI comorbidities
		341	Rome IV	GMM	5		constipation‐predominant; diarrhea‐pain‐predominant; mixed‐high psychological symptoms; mixed‐moderate psychological symptoms; overall mild symptoms
Han et al.^9^	2019	332	Rome II and Rome III	Factor analysis and Latent class Analysis	4	six groups of daily diary symptoms, cognitive beliefs about IBS, and IBS quality of life [QOL]	low symptoms and good QOL, low symptoms and moderate QOL; high symptoms with diarrhea and poor QOL; high symptoms with low diarrhea and moderate QOL
Black et al.^20^	2020	1080	Rome III	Latent class Analysis (LCA)	7	Lower GI, Extra intestinal symptom, GI symptom-specific anxiety, and stress	Constipation-pain with high psychological; constipation with high psychological, diarrhea –pain with low/high psychological; mixed GI with high psychological; overall mild GI severity. with low/high psychological; high GI symptoms and high psychological comorbidity
		811	Rome IV	LCA	7		
Current study	2022	988	Rome III	GUDMM-S	9	Lower and upper GI symptoms, Neo FFI Personality factors, score of anxiety, depression, and somatic symptoms	High Diarrhea-pain and psychological; high upper GI and psychological; moderate overall GI- high psychological; high constipation-high psychological; moderate constipation-low psychological; high diarrhea-high depression; moderate diarrhea-low psychological; overall mild; moderate lower GI-low psychological

Considering the high level of overlap between IBS and other DGBI, in the current study, we included upper GI symptoms that were previously identified associated with IBS^44,45^, in addition to the lower GI. Previous studies on clustering IBS individuals did not thoroughly investigate all these symptoms, but we showed their importance in discriminating between different IBS subgroups.

Although Whitehead et al. declared that there is no unique association between extra-intestinal symptom-based disorders and IBS^46^, the abovementioned studies considered most extra-intestinal somatic symptoms in their clustering. In an experiment, we examined the inclusion of 14 somatic symptoms into our clustering procedure but did not find a significant change in the profile of clusters obtained in their absence. However, we also considered the sum of the frequency of all somatic symptoms, which had a high correlation with depression and anxiety scores in all IBS patients (ρ = 0.49, 0.52).

Cluster 2 was the only cluster where the somatic symptom score was notably higher than psychological factors, which indicated the predominance of upper GI symptoms alongside moderate values of lower GI symptoms.

The application of a machine learning approach with the capability of considering the dependencies of variables on each other in similarity calculation and identifying reproducible sub-groups of IBS patients based on multiple features from various aspects of upper GI, lower GI, personality traits, psychological, and somatic scores is the main strength of the current study. However, due to the report of the greater levels of symptoms by individuals in patient-based studies and the stricter criteria of Rome IV, the population-based nature of the current study and the utilization of the Rome III inclusion criteria could be highlighted as the limitations of the study.

In the present investigation, we have observed some degree of overlap with the Rome III subtypes, and the inclusion of a wider array of symptoms has led to a more distinct categorization of patients. Nevertheless, the clinical implications of these findings remain uncertain at this point. These types of studies serve as the initial phase in a multi-level approach, with the primary goal of analyzing the clinical presentation in IBS patients and identifying clinically meaningful subgroups. In the subsequent phase, further studies will introduce additional investigative levels, including the assessment of responses to therapies, consideration of pathophysiological aspects, and exploration of genotypic characteristics in the identified subgroups.

Different research examining subgroups of IBS might provide different results depending on the utilized clustering algorithms and the investigated factors. However, recent research on this topic has shown promising results, confirming the presence of subgroups of IBS patients with varying degrees of GI and psychological variables. More thorough research, such as considering the starting symptoms (i.e., psychological factors or GI symptoms) or other clinical factors, could also help identify subgroups. This goal in particular, could be accomplished by employing machine learning techniques and giving the important factor more weight. Furthermore, considering the more precise separated groups of patients could help to design more targeted and personalized experiments for investigating the pathophysiological factors.

### Supplementary Information

Supplementary Information.

## Data Availability

The python implementation that supports the findings of this study are available from the corresponding author upon reasonable request.
