# Supplementary material for: Exploring new subgroups for irritable bowel syndrome using a machine learning algorithm
Source: Sci Rep. 2023 Oct 28;13:18483. doi: 10.1038/s41598-023-45605-2 (PMC10613279; doi:10.1038/s41598-023-45605-2)
Supplement: Supplementary file 1 — Supplementary Information. [file 41598_2023_45605_MOESM1_ESM.docx]

Exploring New Subgroups for Irritable Bowel Syndrome Using a Machine Learning Algorithm

Elahe Mousavi, Ammar Hasanzadeh Keshteli, Mohammadreza Sehhati,

Ahmad Vaez, Peyman Adibi

# Supplementary Figures

Supplementary Figure 1. Plot of clustering validation indices, CVNN and S-Dbw, for various number of clusters.


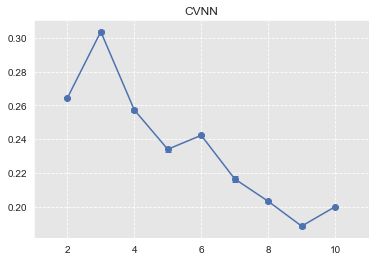

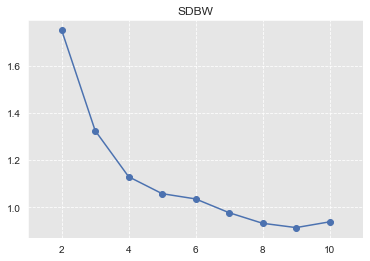

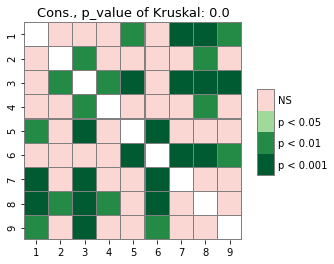

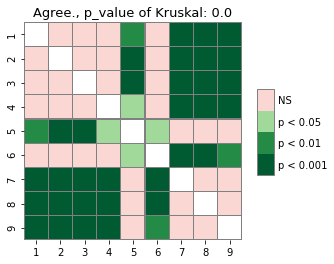

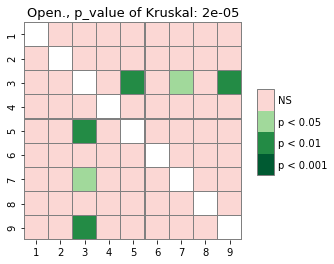

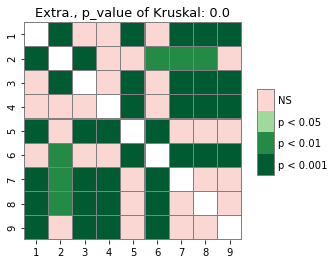

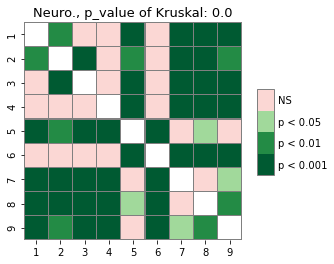

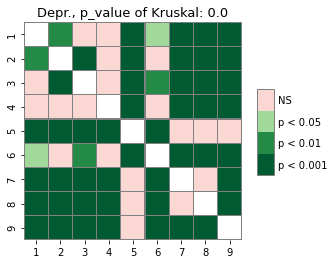

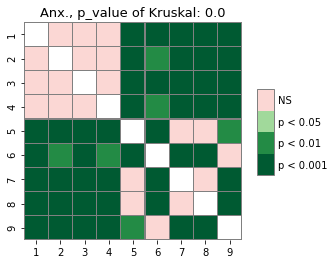

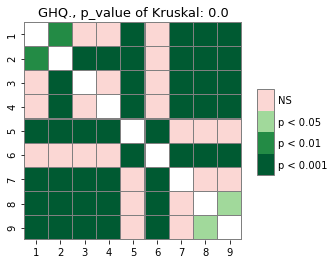

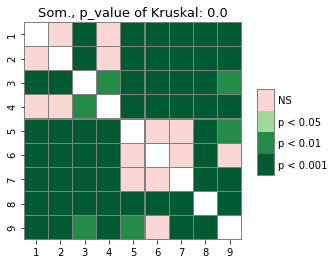

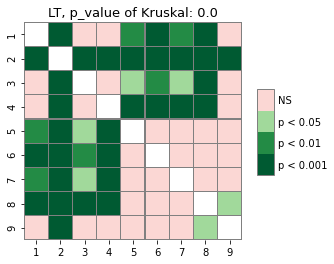

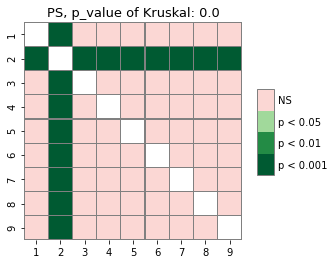

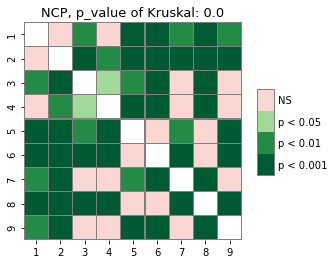

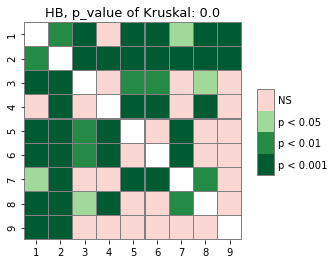

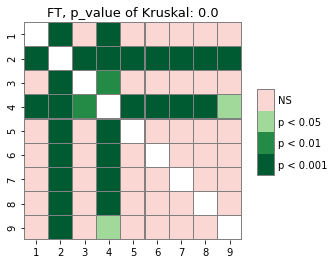

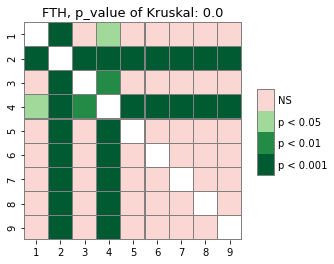

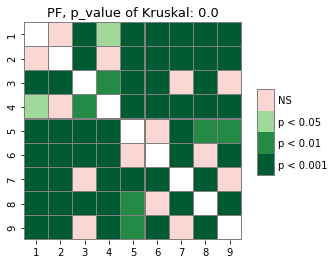

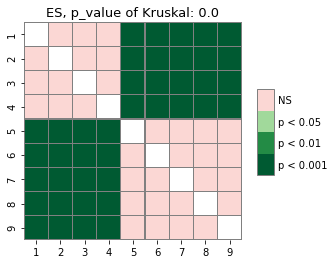

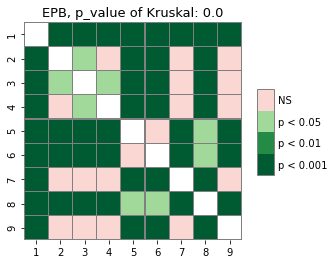

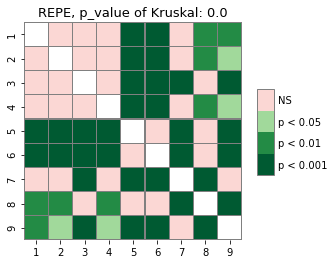

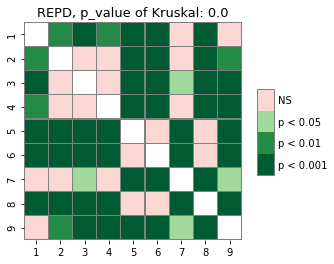

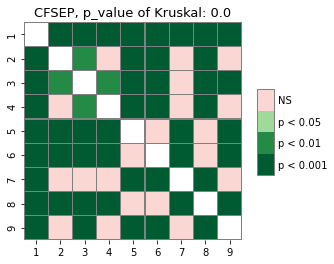

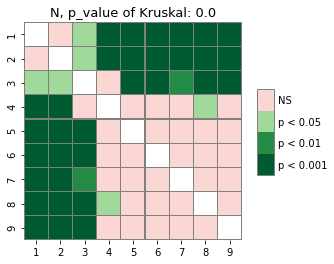

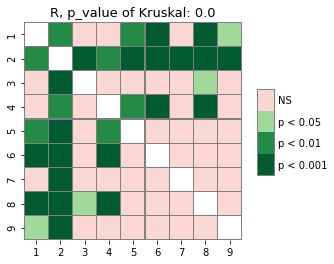

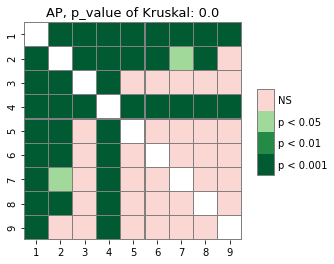


Supplementary Figure 2. Post hoc analysis (Conover and Holm correction) of input variables in 9 identified clusters.


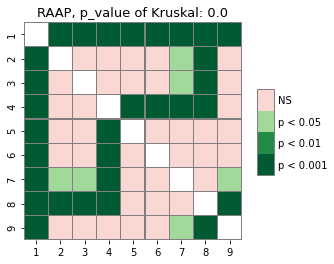

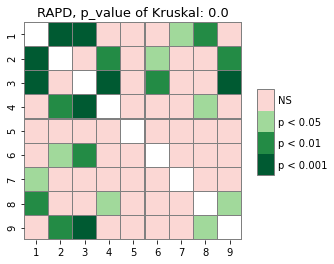

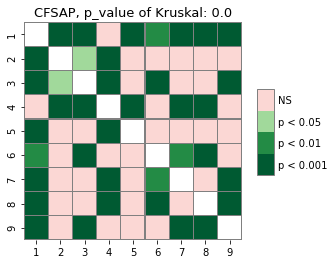

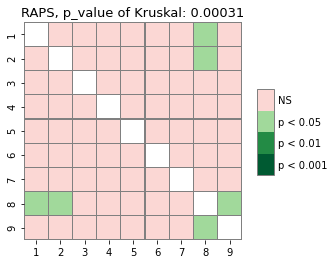

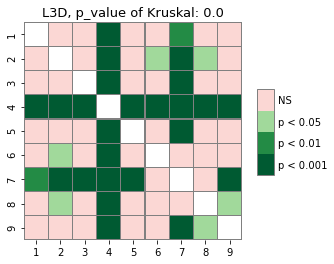

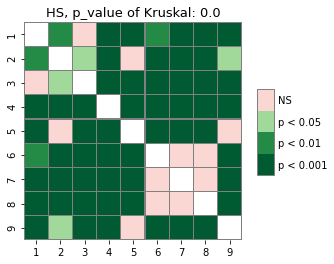

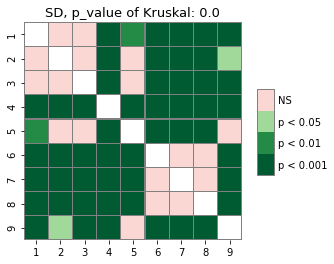

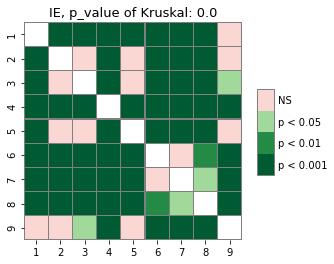

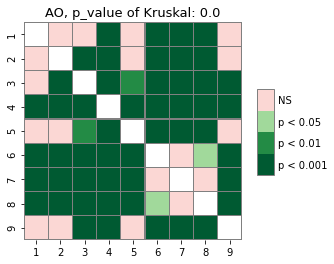

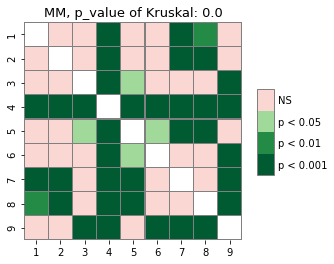

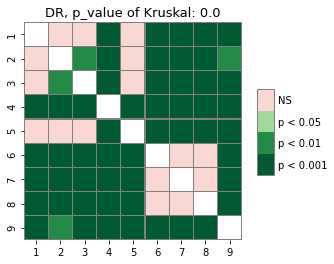

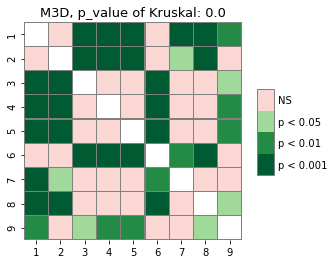

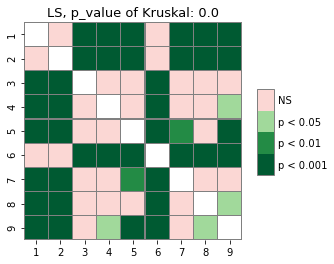

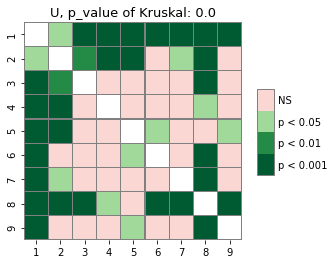

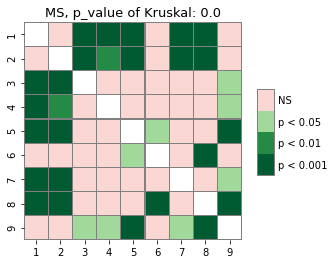

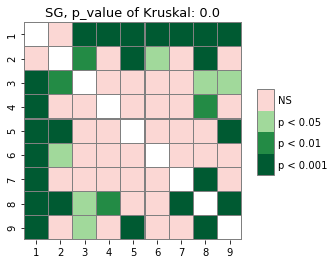

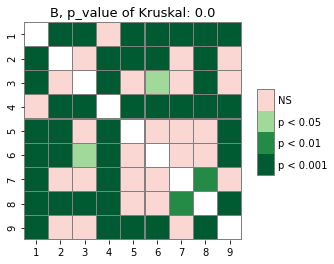

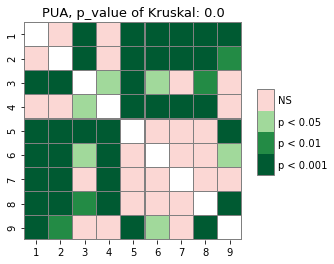

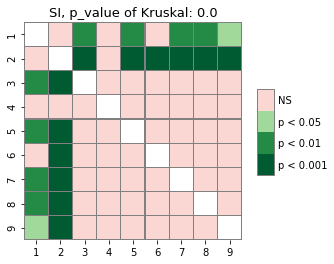

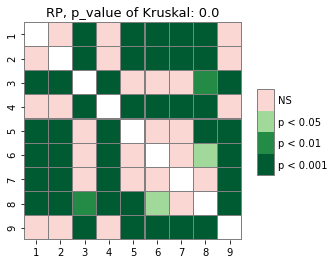

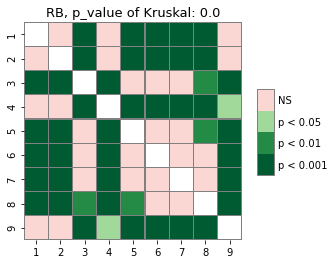


Supplementary Figure 3. (Continued).

Supplementary Figure 4. The somatic symptom profiles in 9 identified clusters.


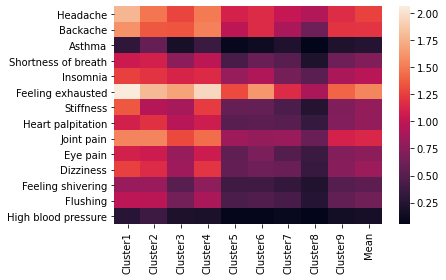


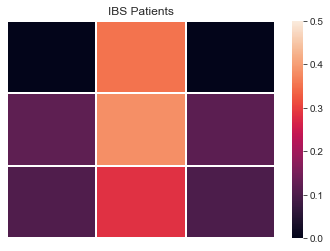

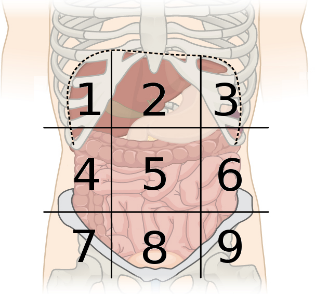

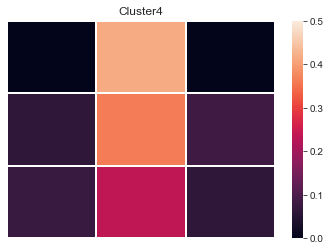

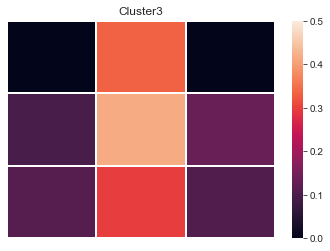

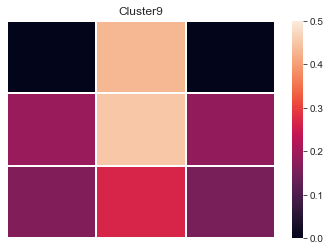

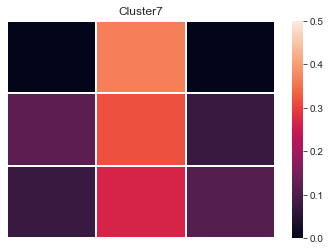

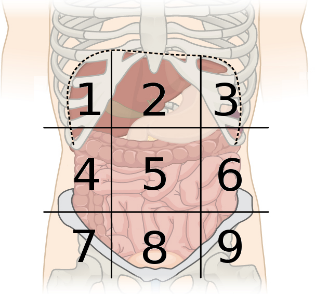

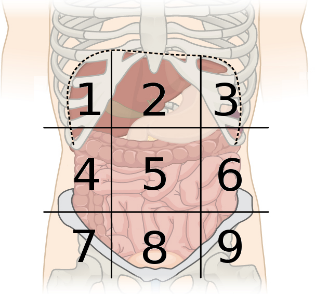

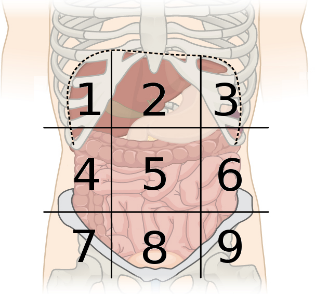

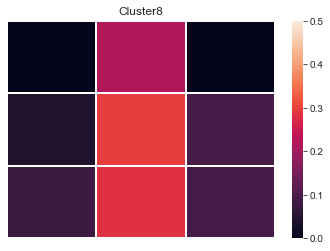

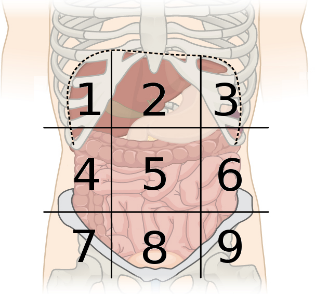

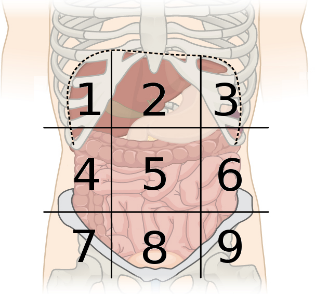

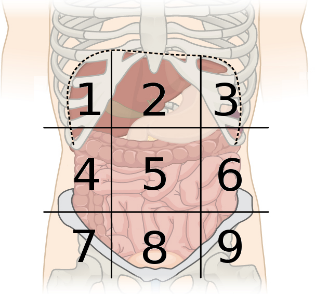

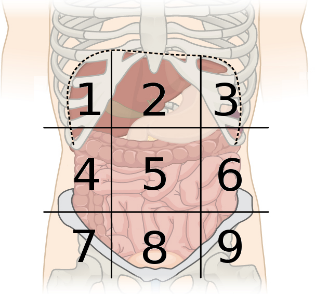

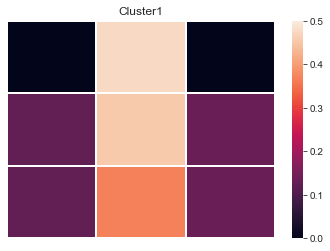

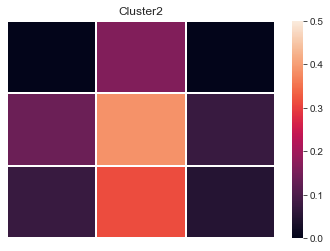

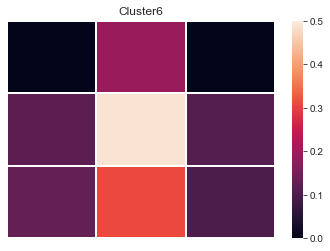

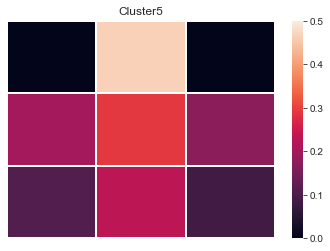

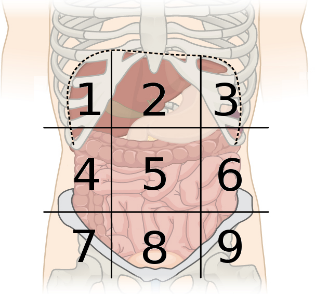

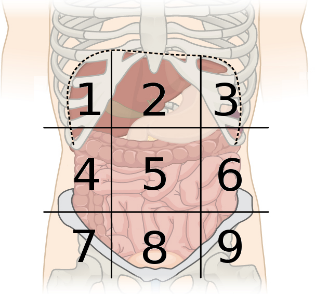

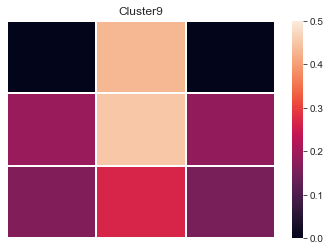


**Cluster 4**

**Cluster 9**

**Cluster 8**

**Cluster 7**

**Cluster 3**

**Cluster 2**

**Cluster 6**

**All of IBS Patients**

**1**

**2**

**3**

**4**

**5**

**6**

**7**

**Cluster 1**

Supplementary Figure 5. Average of segmental abdominal pain including (1) epigastrium, (2) right lumbar, (3) umbilical, (4) left lumbar, (5) right iliac, (6) hypogastrium, (7) left iliac regions in different clusters.

**Cluster 5**

Supplementary Figure 6. Post-hoc analysis of segmental abdominal pain in 9 identified clusters.


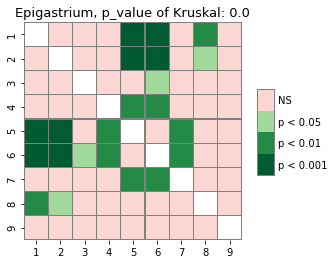

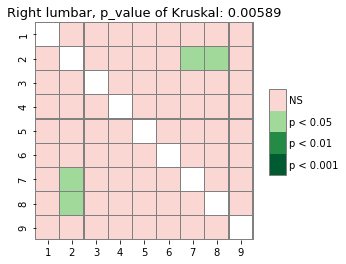


# Supplementary Tables

Supplementary Table 1. Results of pairwise comparison of each identified clusters versus the rest of population for psychological and GI symptoms obtained by Man-Whitney U test, and Cliff’s Delta effect size. The Results of Multiple Comparison Test are obtained by Kruskal-Wallis test.

| Variable | C1 vs. Others | | C2 vs. Others | | C3 vs. Others | | C4 vs. Others | | C5 vs. Others | | C6 vs. Others | | C7 vs. Others | | C8 vs. Others | | C9 vs. Others | | MCT | |
| --- | --- | --- | --- | --- | --- | --- | --- | --- | --- | --- | --- | --- | --- | --- | --- | --- | --- | --- | --- | --- |
|  | **P.val.** | **E.S.** | **P.val.** | **E.S.** | **P.val.** | **E.S.** | **P.val.** | **E.S.** | **P.val.** | **E.S.** | **P.val.** | **E.S.** | **P.val.** | **E.S.** | **P.val.** | **E.S.** | **P.val.** | **E.S.** | **P.val.** | **E.S.** |
| Conscien. | 0.0 | 0.22 | 0.787 | 0.02 | 0.0 | 0.29 | 0.856 | 0.01 | 0.037 | 0.13 | 0.0 | 0.25 | 0.0 | 0.24 | 0.0 | 0.35 | 0.065 | 0.1 | <.0001 | 0.09 |
| Agree. | 0.001 | 0.2 | 0.0 | 0.21 | 0.0 | 0.19 | 0.018 | 0.14 | 0.012 | 0.15 | 0.056 | 0.13 | 0.0 | 0.25 | 0.0 | 0.29 | 0.0 | 0.2 | <.0001 | 0.04 |
| Openness | 0.209 | 0.08 | 0.199 | 0.08 | 0.001 | 0.16 | 0.86 | 0.01 | 0.002 | 0.19 | 0.103 | 0.11 | 0.037 | 0.11 | 0.241 | 0.08 | 0.003 | 0.16 | <.0001 | 0.04 |
| Extra. | 0.0 | **0.3** | 0.563 | 0.03 | 0.0 | **0.4** | 0.004 | 0.17 | 0.0 | 0.23 | 0.0 | 0.29 | 0.0 | 0.35 | 0.0 | 0.38 | 0.0 | 0.25 | <.0001 | **0.2** |
| Neuro. | 0.0 | **0.33** | 0.394 | 0.05 | 0.0 | **0.43** | 0.0 | 0.27 | 0.0 | 0.25 | 0.001 | 0.23 | 0.0 | 0.45 | 0.0 | 0.49 | 0.0 | 0.22 | <.0001 | **0.26** |
| Depression | 0.0 | **0.39** | 0.019 | 0.14 | 0.0 | **0.44** | 0.0 | **0.34** | 0.0 | 0.35 | 0.04 | 0.14 | 0.0 | 0.54 | 0.0 | 0.5 | 0.0 | 0.2 | <.0001 | **0.32** |
| Anxiety | 0.0 | **0.43** | 0.0 | 0.25 | 0.0 | **0.46** | 0.0 | **0.27** | 0.0 | 0.4 | 0.764 | 0.02 | 0.0 | 0.46 | 0.0 | 0.6 | 0.002 | 0.17 | <.0001 | **0.34** |
| GHQ | 0.0 | **0.36** | 0.373 | 0.05 | 0.0 | **0.4** | 0.0 | **0.36** | 0.0 | 0.33 | 0.014 | 0.16 | 0.0 | 0.39 | 0.0 | 0.47 | 0.0 | 0.25 | <.0001 | **0.27** |
| Som. | 0.0 | **0.53** | 0.0 | **0.45** | 0.001 | 0.17 | 0.0 | **0.38** | 0.0 | 0.34 | 0.005 | 0.19 | 0.0 | 0.41 | 0.0 | 0.67 | 0.076 | 0.09 | <.0001 | **0.35** |
| LT | 0.044 | 0.1 | 0.0 | **0.48** | 0.415 | 0.03 | 0.012 | 0.12 | 0.001 | 0.16 | 0.0 | 0.22 | 0.001 | 0.15 | 0.0 | 0.25 | 0.433 | 0.03 | <.0001 | 0.15 |
| PS | 0.094 | 0.07 | 0.0 | **0.51** | 0.678 | 0.01 | 0.908 | 0.0 | 0.307 | 0.04 | 0.001 | 0.15 | 0.001 | 0.13 | 0.007 | 0.13 | 0.808 | 0.01 | <.0001 | 0.17 |
| NCP | 0.0 | 0.25 | 0.0 | **0.44** | 0.701 | 0.02 | 0.001 | 0.18 | 0.0 | 0.27 | 0.0 | 0.33 | 0.859 | 0.01 | 0.0 | 0.44 | 0.771 | 0.01 | <.0001 | 0.18 |
| HB | 0.0 | 0.23 | 0.0 | **0.48** | 0.347 | 0.04 | 0.092 | 0.09 | 0.0 | 0.27 | 0.0 | 0.28 | 0.586 | 0.03 | 0.0 | 0.25 | 0.09 | 0.08 | <.0001 | 0.16 |
| FT | 0.015 | 0.11 | 0.0 | **0.76** | 0.037 | 0.08 | 0.004 | 0.13 | 0.0 | 0.17 | 0.013 | 0.13 | 0.0 | 0.14 | 0.0 | 0.21 | 0.186 | 0.05 | <.0001 | **0.32** |
| FTH | 0.104 | 0.06 | 0.0 | **0.65** | 0.007 | 0.08 | 0.052 | 0.07 | 0.003 | 0.1 | 0.001 | 0.13 | 0.002 | 0.09 | 0.001 | 0.13 | 0.0 | 0.11 | <.0001 | **0.4** |
| PF | 0.0 | **0.48** | 0.0 | **0.4** | 0.401 | 0.04 | 0.0 | 0.27 | 0.0 | 0.3 | 0.0 | 0.47 | 0.762 | 0.02 | 0.0 | 0.58 | 0.121 | 0.08 | <.0001 | **0.3** |
| ES | 0.0 | **0.35** | 0.0 | **0.35** | 0.0 | 0.21 | 0.0 | 0.25 | 0.0 | 0.2 | 0.0 | 0.22 | 0.0 | 0.22 | 0.0 | 0.41 | 0.0 | 0.23 | <.0001 | **0.21** |
| EPB | 0.0 | **0.52** | 0.0 | 0.27 | 0.028 | 0.1 | 0.0 | 0.27 | 0.0 | 0.67 | 0.0 | 0.66 | 0.01 | 0.13 | 0.0 | 0.44 | 0.02 | 0.11 | <.0001 | **0.43** |
| REPE | 0.451 | 0.04 | 0.138 | 0.08 | 0.405 | 0.04 | 0.148 | 0.08 | 0.0 | 0.4 | 0.0 | 0.38 | 0.0 | 0.25 | 0.0 | 0.24 | 0.0 | 0.33 | <.0001 | 0.16 |
| REPD | 0.0 | **0.36** | 0.058 | 0.11 | 0.855 | 0.01 | 0.155 | 0.08 | 0.0 | 0.56 | 0.0 | 0.49 | 0.0 | 0.19 | 0.0 | 0.45 | 0.0 | 0.39 | <.0001 | **0.29** |
| CFSEP | 0.0 | **0.54** | 0.001 | 0.19 | 0.424 | 0.04 | 0.001 | 0.19 | 0.0 | 0.64 | 0.0 | 0.47 | 0.01 | 0.13 | 0.0 | 0.49 | 0.0 | 0.27 | <.0001 | **0.35** |
| N | 0.0 | **0.35** | 0.0 | **0.35** | 0.001 | 0.15 | 0.529 | 0.03 | 0.0 | 0.2 | 0.0 | 0.23 | 0.023 | 0.11 | 0.0 | 0.29 | 0.007 | 0.12 | <.0001 | 0.15 |
| R | 0.008 | 0.14 | 0.0 | **0.41** | 0.957 | 0.0 | 0.018 | 0.12 | 0.002 | 0.16 | 0.0 | 0.21 | 0.409 | 0.04 | 0.0 | 0.25 | 0.092 | 0.08 | <.0001 | 0.11 |
| AP | 0.0 | **0.66** | 0.134 | 0.07 | 0.0 | 0.21 | 0.0 | **0.34** | 0.0 | 0.19 | 0.0 | 0.18 | 0.004 | 0.12 | 0.0 | 0.23 | 0.041 | 0.08 | <.0001 | **0.31** |
| RDAAP | 0.0 | **0.53** | 0.458 | 0.04 | 0.687 | 0.02 | 0.0 | 0.21 | 0.006 | 0.16 | 0.003 | 0.19 | 0.0 | 0.21 | 0.0 | 0.38 | 0.605 | 0.03 | <.0001 | 0.16 |
| RAPD | 0.0 | 0.19 | 0.004 | 0.15 | 0.0 | 0.18 | 0.005 | 0.14 | 0.542 | 0.03 | 0.044 | 0.12 | 0.478 | 0.03 | 0.032 | 0.13 | 0.01 | 0.12 | <.0001 | 0.06 |
| CFSAP | 0.0 | **0.4** | 0.263 | 0.05 | 0.0 | 0.26 | 0.0 | 0.25 | 0.09 | 0.08 | 0.05 | 0.11 | 0.0 | 0.16 | 0.0 | 0.21 | 0.016 | 0.1 | <.0001 | 0.16 |
| RAPCS | 0.04 | 0.11 | 0.055 | 0.1 | 0.095 | 0.08 | 0.306 | 0.06 | 0.311 | 0.06 | 0.081 | 0.11 | 0.047 | 0.1 | 0.003 | 0.18 | 0.041 | 0.1 | <.0001 | 0.03 |
| L3DW | 0.198 | 0.06 | 0.131 | 0.08 | 0.73 | 0.01 | 0.0 | **0.54** | 0.688 | 0.02 | 0.005 | 0.16 | 0.0 | 0.32 | 0.002 | 0.18 | 0.291 | 0.05 | <.0001 | 0.17 |
| LHS | 0.028 | 0.12 | 0.062 | 0.1 | 0.068 | 0.08 | 0.0 | **0.69** | 0.0 | 0.2 | 0.0 | 0.35 | 0.0 | 0.4 | 0.0 | 0.44 | 0.0 | 0.28 | <.0001 | **0.33** |
| SDD | 0.09 | 0.09 | 0.221 | 0.07 | 0.66 | 0.02 | 0.0 | **0.85** | 0.01 | 0.14 | 0.0 | 0.39 | 0.0 | 0.51 | 0.0 | 0.48 | 0.0 | 0.25 | <.0001 | **0.43** |
| SIE | 0.0 | 0.26 | 0.89 | 0.01 | 0.596 | 0.02 | 0.0 | **0.83** | 0.84 | 0.01 | 0.0 | 0.34 | 0.0 | 0.43 | 0.0 | 0.6 | 0.003 | 0.15 | <.0001 | **0.42** |
| AO | 0.126 | 0.08 | 0.002 | 0.17 | 0.21 | 0.06 | 0.0 | **0.86** | 0.01 | 0.15 | 0.0 | 0.4 | 0.0 | 0.5 | 0.0 | 0.6 | 0.001 | 0.17 | <.0001 | **0.46** |
| MM | 0.941 | 0.0 | 0.579 | 0.03 | 0.002 | 0.13 | 0.0 | **0.56** | 0.197 | 0.06 | 0.002 | 0.17 | 0.0 | 0.29 | 0.0 | 0.27 | 0.0 | 0.18 | <.0001 | **0.21** |
| DR | 0.981 | 0.0 | 0.015 | 0.13 | 0.114 | 0.07 | 0.0 | **0.78** | 0.455 | 0.04 | 0.0 | 0.41 | 0.0 | 0.48 | 0.0 | 0.49 | 0.0 | 0.34 | <.0001 | **0.42** |
| M3D | 0.0 | **0.31** | 0.001 | 0.17 | 0.001 | 0.14 | 0.0 | 0.19 | 0.0 | 0.19 | 0.0 | **0.24** | 0.344 | 0.04 | 0.003 | 0.18 | 0.171 | 0.06 | <.0001 | 0.11 |
| LS | 0.0 | **0.32** | 0.0 | **0.28** | 0.003 | 0.13 | 0.0 | 0.2 | 0.0 | 0.27 | 0.0 | **0.31** | 0.53 | 0.03 | 0.0 | 0.21 | 0.973 | 0.0 | <.0001 | 0.15 |
| U | 0.0 | **0.43** | 0.0 | 0.2 | 0.296 | 0.05 | 0.037 | 0.11 | 0.0 | 0.2 | 0.35 | 0.06 | 0.313 | 0.05 | 0.0 | 0.37 | 0.496 | 0.03 | <.0001 | 0.13 |
| MS | 0.0 | **0.26** | 0.0 | 0.18 | 0.041 | 0.08 | 0.07 | 0.09 | 0.001 | 0.16 | 0.136 | 0.08 | 0.037 | 0.09 | 0.0 | 0.24 | 0.008 | 0.11 | <.0001 | 0.09 |
| SG | 0.0 | **0.41** | 0.001 | 0.18 | 0.031 | 0.1 | 0.652 | 0.02 | 0.0 | 0.22 | 0.098 | 0.1 | 0.748 | 0.02 | 0.0 | 0.33 | 0.024 | 0.11 | <.0001 | 0.12 |
| B | 0.0 | **0.6** | 0.388 | 0.05 | 0.046 | 0.09 | 0.0 | **0.43** | 0.0 | 0.28 | 0.0 | 0.32 | 0.018 | 0.12 | 0.0 | 0.39 | 0.88 | 0.01 | <.0001 | **0.25** |
| PUA | 0.0 | **0.36** | 0.0 | 0.26 | 0.925 | 0.0 | 0.0 | 0.18 | 0.0 | 0.3 | 0.0 | 0.21 | 0.005 | 0.13 | 0.0 | 0.27 | 0.668 | 0.02 | <.0001 | 0.16 |
| SI | 0.001 | 0.09 | 0.0 | 0.16 | 0.047 | 0.04 | 0.09 | 0.04 | 0.044 | 0.05 | 0.256 | 0.03 | 0.026 | 0.05 | 0.053 | 0.06 | 0.127 | 0.04 | <.0001 | 0.07 |
| RP | 0.0 | 0.23 | 0.0 | 0.33 | 0.0 | 0.18 | 0.0 | 0.34 | 0.024 | 0.12 | 0.004 | 0.17 | 0.0 | 0.26 | 0.0 | 0.43 | 0.0 | 0.19 | <.0001 | **0.2** |
| RB | 0.0 | 0.2 | 0.0 | 0.29 | 0.001 | 0.15 | 0.0 | **0.4** | 0.059 | 0.1 | 0.0 | 0.22 | 0.0 | 0.26 | 0.0 | 0.41 | 0.0 | 0.19 | <.0001 | **0.19** |

Supplementary Table 2. Results of pairwise comparison of somatic symptoms for each identified clusters versus the rest of population obtained by Man-Whitney U test, and Cliff’s Delta effect size (ES). The Results of Multiple Comparison Test (MCT) are obtained by Kruskal-Wallis test.

|  | C1 vs. others | | C2 vs. others | | C3 vs. others | | C4 vs. others | | C5 vs. others | | C6 vs. others | | C7 vs. others | | C8 vs. others | | C9 vs. others | | MCT | |
| --- | --- | --- | --- | --- | --- | --- | --- | --- | --- | --- | --- | --- | --- | --- | --- | --- | --- | --- | --- | --- |
|  | **P.val.** | **ES** | **P.val.** | **ES** | **P.val.** | **ES** | **P.val.** | **ES** | **P.val.** | **ES** | **P.val.** | **ES** | **P.val.** | **ES** | **P.val.** | **ES** | **P.val.** | **ES** | **P.val.** | **ES** |
| Headache | 0.0 | 0.38 | 0.002 | 0.16 | 0.919 | 0.0 | 0.002 | 0.17 | 0.025 | 0.12 | 0.218 | 0.07 | 0.0 | 0.2 | 0.0 | 0.26 | 0.105 | 0.08 | 0.0 | 0.105 |
| Backache | 0.0 | 0.28 | 0.018 | 0.13 | 0.022 | 0.1 | 0.0 | 0.22 | 0.011 | 0.14 | 0.747 | 0.02 | 0.0 | 0.25 | 0.0 | 0.42 | 0.926 | 0.0 | 0.0 | 0.117 |
| Asthma | 0.112 | 0.07 | 0.0 | 0.28 | 0.156 | 0.05 | 0.115 | 0.06 | 0.003 | 0.13 | 0.032 | 0.1 | 0.701 | 0.01 | 0.002 | 0.15 | 0.824 | 0.01 | 0.0 | 0.07 |
| Shortness of breath | 0.0 | 0.23 | 0.0 | 0.31 | 0.1 | 0.07 | 0.001 | 0.17 | 0.0 | 0.22 | 0.291 | 0.06 | 0.001 | 0.16 | 0.0 | 0.41 | 0.32 | 0.05 | 0.0 | 0.124 |
| Insomnia | 0.0 | 0.23 | 0.0 | 0.19 | 0.001 | 0.14 | 0.017 | 0.13 | 0.033 | 0.12 | 0.935 | 0.0 | 0.0 | 0.26 | 0.0 | 0.35 | 0.273 | 0.05 | 0.0 | 0.103 |
| Feeling exhausted | 0.0 | 0.4 | 0.0 | 0.2 | 0.021 | 0.11 | 0.0 | 0.3 | 0.001 | 0.19 | 0.28 | 0.07 | 0.0 | 0.32 | 0.0 | 0.49 | 0.015 | 0.12 | 0.0 | 0.194 |
| Stiffness | 0.0 | 0.38 | 0.01 | 0.14 | 0.022 | 0.11 | 0.0 | 0.27 | 0.013 | 0.14 | 0.014 | 0.15 | 0.0 | 0.26 | 0.0 | 0.41 | 0.355 | 0.05 | 0.0 | 0.149 |
| Heart palpitation | 0.0 | 0.24 | 0.0 | 0.33 | 0.002 | 0.14 | 0.0 | 0.21 | 0.0 | 0.23 | 0.0 | 0.22 | 0.0 | 0.23 | 0.0 | 0.37 | 0.608 | 0.03 | 0.0 | 0.15 |
| joint pain | 0.0 | 0.28 | 0.0 | 0.29 | 0.007 | 0.13 | 0.001 | 0.19 | 0.0 | 0.2 | 0.0 | 0.22 | 0.0 | 0.23 | 0.0 | 0.36 | 0.86 | 0.01 | 0.0 | 0.134 |
| Eye pain | 0.0 | 0.24 | 0.0 | 0.22 | 0.345 | 0.04 | 0.003 | 0.16 | 0.011 | 0.14 | 0.357 | 0.06 | 0.0 | 0.22 | 0.0 | 0.31 | 0.969 | 0.0 | 0.0 | 0.085 |
| Dizziness | 0.0 | 0.31 | 0.0 | 0.28 | 0.529 | 0.03 | 0.0 | 0.31 | 0.0 | 0.21 | 0.009 | 0.16 | 0.0 | 0.2 | 0.0 | 0.4 | 0.301 | 0.05 | 0.0 | 0.156 |
| Feeling shivering | 0.0 | 0.24 | 0.0 | 0.26 | 0.762 | 0.01 | 0.002 | 0.16 | 0.022 | 0.12 | 0.026 | 0.13 | 0.0 | 0.2 | 0.0 | 0.27 | 0.841 | 0.01 | 0.0 | 0.091 |
| Flushing | 0.0 | 0.22 | 0.0 | 0.3 | 0.444 | 0.03 | 0.001 | 0.19 | 0.004 | 0.16 | 0.016 | 0.15 | 0.001 | 0.17 | 0.0 | 0.31 | 0.479 | 0.03 | 0.0 | 0.101 |
| High blood pressure | 0.039 | 0.07 | 0.0 | 0.15 | 0.085 | 0.05 | 0.571 | 0.02 | 0.008 | 0.1 | 0.042 | 0.08 | 0.051 | 0.06 | 0.027 | 0.09 | 0.66 | 0.01 | 0.0 | 0.042 |

Supplementary Table 3. Kruskal-Wallis test for segmental abdominal pain.

| Place No. | 1 | 2 | 3 | 4 | 5 | 6 | 7 |
| --- | --- | --- | --- | --- | --- | --- | --- |
| P-Value | <0.001 | 0.0058 | 0.015 | 0.078 | 0.352 | 0.463 | 0.291 |
